# Dual RNA-Seq transcriptome analysis of chicken macrophage-like cells (HD11) infected *in vitro* with *Eimeria tenella*

**DOI:** 10.1017/S0031182021000111

**Published:** 2021-05

**Authors:** Arnar K. S. Sandholt, Feifei Xu, Robert Söderlund, Anna Lundén, Karin Troell, Staffan G. Svärd, Eva Wattrang

**Affiliations:** 1 Department of Microbiology, National Veterinary Institute, SE-751 89 Uppsala, Sweden; 2 Department of Cell and Molecular Biology, BMC, Uppsala University, Box 596, SE-751 24 Uppsala, Sweden

**Keywords:** Chicken, coccidiosis, dual RNA-Seq, *Eimeria tenella*, infection biology, transcriptome analysis

## Abstract

The study aimed to monitor parasite and host gene expression during the early stages of *Eimeria tenella* infection of chicken cells using dual RNA-Seq analysis. For this, we used chicken macrophage-like cell line HD11 cultures infected *in vitro* with purified *E. tenella* sporozoites. Cultures were harvested between 2 and 72 h post-infection and mRNA was extracted and sequenced. Dual RNA-Seq analysis showed clear patterns of altered expression for both parasite and host genes during infection. For example, genes in the chicken immune system showed upregulation early (2–4 h), a strong downregulation of genes across the immune system at 24 h and a repetition of early patterns at 72 h, indicating that invasion by a second generation of parasites was occurring. The observed downregulation may be due to immune self-regulation or to immune evasive mechanisms exerted by *E. tenella*. Results also suggested pathogen recognition receptors involved in *E. tenella* innate recognition, MRC2, TLR15 and NLRC5 and showed distinct chemokine and cytokine induction patterns. Moreover, the expression of several functional categories of *Eimeria* genes, such as rhoptry kinase genes and microneme genes, were also examined, showing distinctive differences which were expressed in sporozoites and merozoites.

## Graphical abstract

**Figure:**
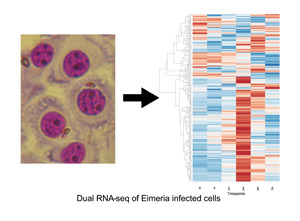

## Introduction

In all types of modern poultry husbandry, coccidiosis is one of the most economically important infectious diseases (Chapman *et al*., 2013; Blake and Tomley, 2014; Witcombe and Smith, 2014). The disease is caused by obligate intracellular protozoan parasites of the genus *Eimeria*, phylum Apicomplexa, and seven different *Eimeria* species can infect domestic fowl. The life cycle of *Eimeria* is monoxenous and involves three phases: sporulation of oocysts that occur outside the host, schizogony (repeated asexual replication over several generations) and gametogony (sexual replication) that occur inside the host. The oocyst stage is extremely resilient in the environment and to chemical disinfectants and the infection is therefore very difficult to eliminate solely with hygienic measures. Currently, disease control in poultry relies on either routine chemoprophylaxis using so-called coccidiostats or vaccination with live virulent or attenuated *Eimeria* parasites (Witcombe and Smith, 2014; Soutter *et al*., 2020). Both methods work satisfactory but are not sustainable due to several reasons including the development of parasite resistance against coccidiostats, difficulties in scaling up production of live vaccines, costs and ethics. The demand for new control measures such as a subunit vaccine is therefore high. However, in order to effectively develop and evaluate new prophylactic methods more knowledge on infection biology and parasite–host interaction between *Eimeria* and the chicken is needed. It is known that *Eimeria* infection results in robust *Eimeria* species-specific immunity in chickens and that features associated with Th1-type responses such as cytotoxic T-cells and *IFN-γ* production rather than specific antibody development are important in protective immunity (McDonald and Shirley, 2009; Kim *et al*., 2019; Soutter *et al*., 2020). Nonetheless, information on the early recognition of *Eimeria* infection by host cells and initiation of ensuing immune responses as well as on parasite activities during this stage is very limited. For example, a crucial event in the initiation of protective immune responses is the correct recognition of pathogen-associated molecular patterns (PAMPs) by pathogen recognition receptors (PRR) of the innate immune system. While PRR recognition of bacterial and viral infections (Kawai and Akira, 2009) has been more intensely studied, their involvement in recognition of Apicomplexan protozoa has also been described (Denkers, 2010). Also, protozoan PAMPs (Egan *et al*., 2009; Ghosh and Stumhofer, 2013; Dos-Santos *et al*., 2016), e.g. glycosylphosphatidylinositol (GPI)-anchored surface antigens (SAGs), including those from *E. tenella* (Chow *et al*., 2011) have been suggested. There are also studies that indicate expressional changes for some PRR during acute *Eimeria* infections of chickens (Sumners *et al*., 2011; Zhang *et al*., 2012) and in *Eimeria* stimulated chicken cells *in vitro* (Zhou *et al*., 2013). However, no real consensus on PRR that are involved in the recognition of *Eimeria* parasites nor on the ligands involved has yet been reached.

Host–pathogen interactions during an infection are dependent on complex and interlinked alterations in the gene expression patterns of both parties. Dual RNA-Seq refers to high-throughput sequencing of the transcriptome of both an infected host and a pathogen in the same sample and allows for precise and sensitive characterization of such gene expression pattern changes. Among intracellular parasites, the methodology has been used to study parasite and host cell transcriptomes of e.g. *Trypanosoma cruzi* (Li *et al*., 2016) and *Leishmania* spp (Dillon *et al*., 2015; Fernandes *et al*., 2016) *in vitro*. *In vivo* transcriptional responses of *E. falciformis* (Ehret *et al*., 2017) and *Toxoplasma gondii* (Pittman *et al*., 2014) and their murine hosts have also been studied using dual RNA-Seq. We wanted to explore this approach to obtain a comprehensive view of parasite and host events during the early phase of *Eimeria* infection of chicken cells *in vitro*. *Eimeria* parasites are not readily propagated to perform their full life cycle in cell-line culture but cell-line systems where the first generation schizogony of *E. tenella* takes place have been described (Patton, 1965; Crane *et al*., 1984; Heriveau *et al*., 2000; Tierney and Mulcahy, 2003; Bussière *et al*., 2018). *Eimeria tenella* is one of the most pathogenic *Eimeria* species that infects chickens and it replicates exclusively in the chicken caecal tissues (Chapman and Shirley, 2003). Several cell types, mammalian and avian, can sustain *E. tenella* replication *in vitro* to a degree, but with varying success with respect to the production of free first-generation merozoites. Madin-Darby bovine kidney (MDBK) cells are often referred to as a ‘gold standard’ (Patton, 1965; Crane *et al*., 1984; Heriveau *et al*., 2000; Tierney and Mulcahy, 2003; Bussière *et al*., 2018). Of the immortal chicken cell lines available to us we found that the retrovirus transformed chicken macrophage cell-line HD11 (Beug *et al*., 1979) sustained *E. tenella* replication to the same degree or better than MDBK cells (unpublished data). In this system, we observed first-generation schizonts and some free merozoites by light microscopy at 48–50 h post-infection (hpi), while the major number of free merozoite clusters appeared at 60–72 hpi. This agrees with how this *E. tenella* strain (Houghton) behaves *in vivo* where first-generation schizonts start to appear 48 hpi and are present at maximum numbers at 60 hpi (Chapman and Shirley, 2003).

Thus, to gain more insight into the *Eimeria–*chicken interaction during the first phase of parasite infection we used the *in vitro E. tenella* infection model in HD11 for dual RNA-Seq analysis. The study aimed to monitor the kinetics of the early transcriptional events of the parasites and the chicken host cells with special focus on potential mechanisms of host recognition of parasite infection.

## Materials and methods

### Maintenance of the *E. tenella* isolate and generation of sporulated oocysts

The authors assert that all procedures contributing to this work comply with the ethical standards of the relevant national and institutional guides on the care and use of laboratory animals. A pure *E. tenella* Houghton strain isolate (Chapman and Shirley, 2003) was maintained by twice-yearly passage in chickens, which was approved by the Uppsala Regional Ethical Committee for Animal Experiments, permit no. C44/16, and sporulated oocysts were prepared from feces as previously described (Wattrang *et al*., 2016).

### Isolation and purification of *E. tenella* sporozoites

Sporozoites were purified from sporulated *E. tenella* oocysts stored for a maximum of 1-month at 4°C using a protocol described by Schmatz *et al*. (1984). In brief, sporulated oocysts were surface sterilized by NaClO solution, washed, mechanically disrupted with glass beads after which sporocysts were opened using taurocholic acid and trypsin. Sporozoites were purified using DE-52 anion exchange chromatography matrix (Whatman^®^, Sigma–Aldrich Merck; this is a discontinued product) and counted in 0.4% trypan blue solution and suspended in fetal calf serum (FCS; Gibco^®^ #10082147, ThermoFisher Scientific) with 10% dimethyl sulfoxide and cryopreserved in liquid nitrogen until used for infections in cell culture.

### Infection of HD11 cells with *E. tenella* sporozoites

The immortalized chicken macrophage cell-line HD11 (Beug *et al*., 1979) was maintained in the growth medium, i.e. RPMI 1640 (National Veterinary Institute) supplemented with 200 IU penicillin mL^−1^, 100 *μ*g streptomycin  mL^−1^, 2 mm L-glutamine, and 5% FCS. Before infection HD11 cells were trypsinised and live cells were counted by trypan blue exclusion. Cells were seeded at 5.6 × 10^5^ cells per well in flat-bottomed 6-well tissue culture plates (Nunc^™^, ThermoFisher Scientific) in 2 mL of growth medium and at 0.35 × 10^5^ cells per well in flat-bottomed 96-well tissue culture plates (Nunc^™^, ThermoFisher Scientific) in 100 *μ*L of growth medium. Plates were incubated at 40°C, 5.2% CO_2_ in air at a humid atmosphere for 24 h after which the HD11 cells were approximately 50–70% confluent in the wells. Cryopreserved *E. tenella* sporozoites were thawed into RPMI 1640 medium with 15% FCS, centrifuged down at 910 × ***g*** for 7 min, resuspended in growth medium and viable sporozoites were counted by trypan blue exclusion. At infection, 4.5 × 10^6^ sporozoites per well in 6-well plates and 0.28 × 10^6^ sporozoites per well in 96-well plates were added to the cultures aiming at a ratio of 4 sporozoites per HD11 cell. The same volume of growth medium without parasites was added to uninfected control cultures. Plates were then cultured until RNA harvest at 2, 4, 12, 24, 48 and 72 hpi. For plates cultured longer than 24 h, growth medium was removed at 24 h, cultures washed with phosphate-buffered saline (PBS; without Ca^2+^ and Mg^2+^ at pH 7) to remove loose sporozoites and fresh growth medium was added to the wells before culture was continued. At RNA harvest in 6-well cultures, the growth medium was removed, and cultures were washed gently with PBS, where after 1 mL TRIzol^®^ Reagent (ThermoFisher Scientific) was added to the well. The cells were detached and dissolved by pipetting up and down and the suspension was subsequently frozen at −70°C and stored until RNA isolation. For each time point, both infected and uninfected cultures were harvested in parallel. Pure sporozoites were used as a control for parasites. All controls and samples, except for the pure sporozoite sample, were harvested in triplicates for biological replicates. All sporozoites used in the present study were isolated at one occasion from the same batch of oocysts. For each thawed aliquot of sporozoites 96-well cultures of infected HD11 cells were set up in parallel to the 6-well cultures for RNA harvest and these were monitored by light microscopy for 72 h to observe schizont development and appearance of clusters of live merozoites to ensure that the first schizogony was completed in the cultures.

### RNA isolation

For RNA isolation 1 mL of HD11 cell lysate in TRIzol was used and total RNA was extracted according to the TRIzol manufacturer's protocol. The isolated RNA was subsequently treated with DNase (TURBO^™^ DNase, 2 U *μ*L^−1^, ThermoFisher Scientific) according to the manufacturers' protocol and further purified using reagents and the ‘RNA clean-up’ protocol of the RNeasy Mini kit (Qiagen). RNA concentration and quality were then assessed using the Agilent RNA 6000 Nano kit on a 2100 Bioanalyzer Instrument (Agilent) and the RNA stored at −70°C until further analysis.

### Sequencing

The dataset consisted of samples taken from infected and uninfected chicken cell cultures at 2, 4, 12, 24, 48 and 72 hpi, each in triplicate cultures. The sequencing libraries were prepared from 120 ng and 500 ng total RNA using the TruSeq stranded mRNA library preparation kit (Illumina) including poly-A selection. The sequencing was done with a HiSeq 2500 machine (Illumina) with 125 bp reads using v4 sequencing chemistry. A pilot sequencing of one pair of samples from 2 hpi and two pairs from 4 and 24 hpi were prepared from 500 ng RNA and the rest from 120 ng. The resulting data were deposited in the Gene Expression Omnibus under accession number GSE154393 and the Sequence Read Archive under accession number SRP271757.

### Read counting

The data were quality checked using FastQC v 0.11.8 (Andrews, 2010) with default settings. MultiQC v 1.8 (Ewels *et al*., 2016) was used to collate reports. The raw read data were trimmed using Trimmomatic v 0.36 (Bolger *et al*., 2014), with a sliding window of length four and an average quality threshold of 20, removing Illumina adapter sequences and removing any reads shorter than 50 bp after trimming. The read mapping was done using the STAR mapper v 2.7.2b (Dobin *et al*., 2013) using default settings. The reads were mapped to the concatenated reference genomes for *Gallus gallus* (GCF_000002315.6_GRCg6a) and *Eimeria tenella* (GCF_499545.2_ETH001). The reads mapping to features were counted using HTSeq v 0.9.1 (Anders *et al*., 2015), with strandedness set to reverse and otherwise default settings. The computations were performed on resources provided by SNIC through Uppsala Multidisciplinary Centre for Advanced Computational Science (UPPMAX) under Project SNIC 2020/15–16.

### Differential expression analysis

Differential expression (DE) analysis was run in edgeR v 3.28.1 (Robinson *et al*., 2010) using the Quasi-likelihood F-test (glmQLFTest). The thresholds used for DE was a |log2 fold change| >1 and an FDR, i.e. *P* value adjusted for multiple hypothesis testing using the Benjamini-Hochberg method, of <0.05. The comparisons made were infected *vs* uninfected at each sampling time for chicken data and parasites at the infection timepoints *vs* a pure sporozoite sample for *E. tenella* data. Gene Ontology (GO) and Kyoto Encyclopaedia of Genes and Genomes (KEGG) enrichment analysis of the chicken data was accomplished using the R packages GO.db (Carlson, 2019*a*), org.Gg.eg.db (Carlson, 2019*b*), and the KEGGrest API (Tenenbaum, 2020), along with edgeR functions. Due to a lack of available annotation packages for *E. tenella*, the GO and KEGG annotations were taken from ToxoDB (Gajria *et al*., 2008) and generated through KEGG's BlastKOALA tool, respectively. The enrichment analysis was done using *ad hoc* scripts, available from the authors on request. Visualization of the results was done using the following R packages: EnhancedVolcano (Blighe *et al*., 2020), ggbiplot (Vu, 2011), ggplot2 (Wickham, 2016), ClassDiscovery (Coombes, 2019), RColorBrewer (Neuwirth, 2014) and functions that are part of edgeR. The data used as input were CPM (Counts Per Million) normalized count data from both organisms that had been filtered to exclude genes with low expression across samples.

## Results

### Sequencing and read counting

Sequencing was performed on 39 RNA samples from *E. tenella* infected and uninfected chicken HD11 macrophage cells at 2, 4, 12, 24, 48 and 72 hpi and a pure sample of *E. tenella* sporozoites, all in triplicates except the sporozoite sample. A minimum of 10 million reads was generated from each sample. Read counting was subsequently performed, the reads were mapped to both the chicken and *E. tenella* genomes simultaneously and the number of reads mapping to genes counted. Supplementary Table S1 shows the information on the samples and the fraction of reads mapping to features, i.e. any expressed parts of the genome. The mapping rate to features was generally high for HD11 samples, ~80–85%, but much lower for the pure *E. tenella* sample, ~65%.

**Fig. 1.**
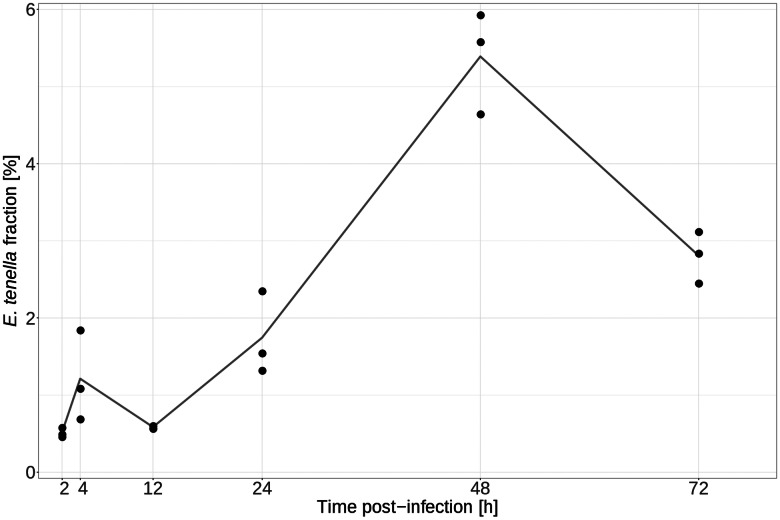
The proportion of *E. tenella* read counts in mRNA samples from chicken HD11 cells infected *in vitro* with purified *E. tenella* sporozoites at 0 h. Black dots represent individual sample values and the line represents mean values at the indicated time points post-infection.

The fraction of reads mapping to the *E. tenella* genome varied considerably (min of 0.466% at 2 hpi and max of 5.936% at 48 hpi) over time (Fig. 1). There was also a considerable variance between samples at certain time points. The average fraction of *E. tenella* reads was only ~0.5% at 12 hpi but reached an average of 5.4% at 48 hpi. It then decreased to approximately 3% at 72 hpi. A potential explanation for this pattern is that at 12 hpi, the parasite is in the early stages of the trophozoite form, where it is not yet dividing. At 48 hpi, the parasite has likely finished several rounds of asexual replication within the first merogony, resulting in a large number of merozoites. At 72 hpi, the first merogony is most likely finished in a large proportion of the infected cells resulting in the release of merozoites, some of which were probably washed away and lost when the RNA was harvested.

### Multidimensional scaling and DE analysis

**Fig. 2.**
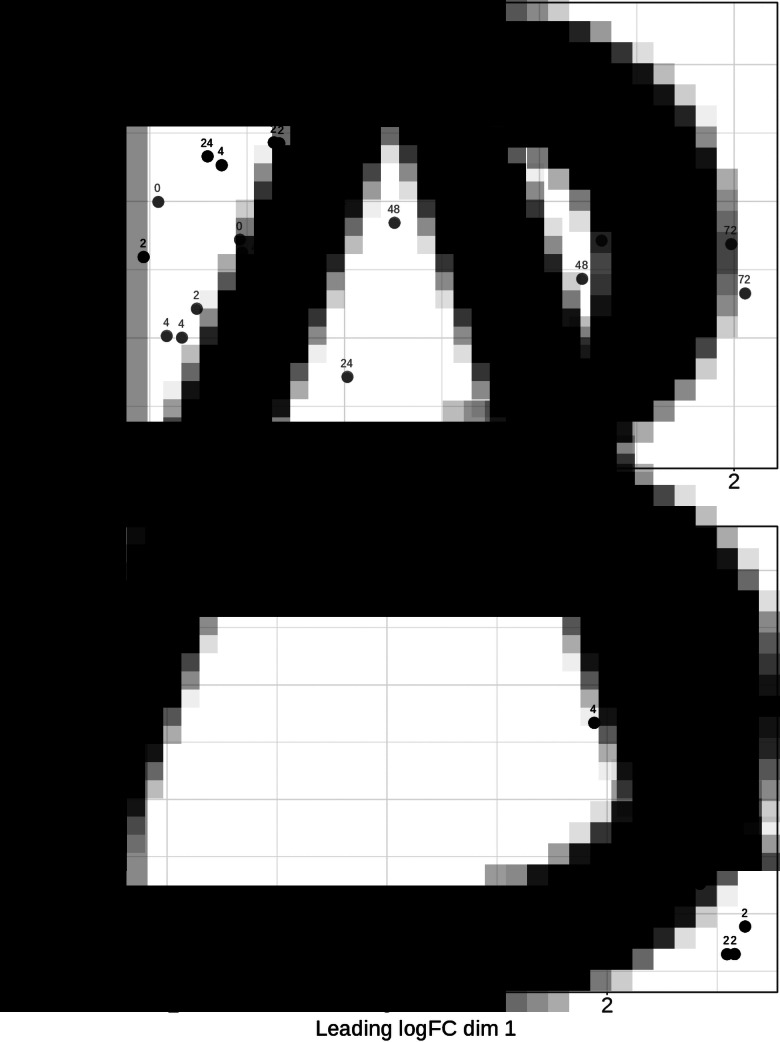
Multidimensional scaling plots for the normalized count data in mRNA samples collected at the indicated time points from uninfected chicken HD11 cells or HD11 cells infected *in vitro* with purified *E. tenella* sporozoites at 0 h. Panel A shows individual sample values for chicken data with infected samples in black and uninfected in grey. Panel B shows individual sample values for *E. tenella* data.

A multidimensional scaling (MDS) analysis was performed on the count data (Fig. 2), revealing a more scattered distribution for the chicken data compared to the *E. tenella* data. For the chicken gene expression, infected and uninfected samples were not clearly separated but had a trend of separation along the axis of leading log fold change 1. A weak trend of separation of early and late time points was evident along the axis of leading log fold change 2. This contrasted dramatically with the *E. tenella* data where the MDS plot showed samples from close time points clustering, with three main clusters forming for 2/4, 12/24 and 48/72 hpi samples. This clustering indicates that *E. tenella* has three distinct stages during the experiment: sporozoite-like at 0–4 hpi, trophozoite-like at 12–24 hpi and merozoite-like at 48–72 hpi.

**Fig. 3.**
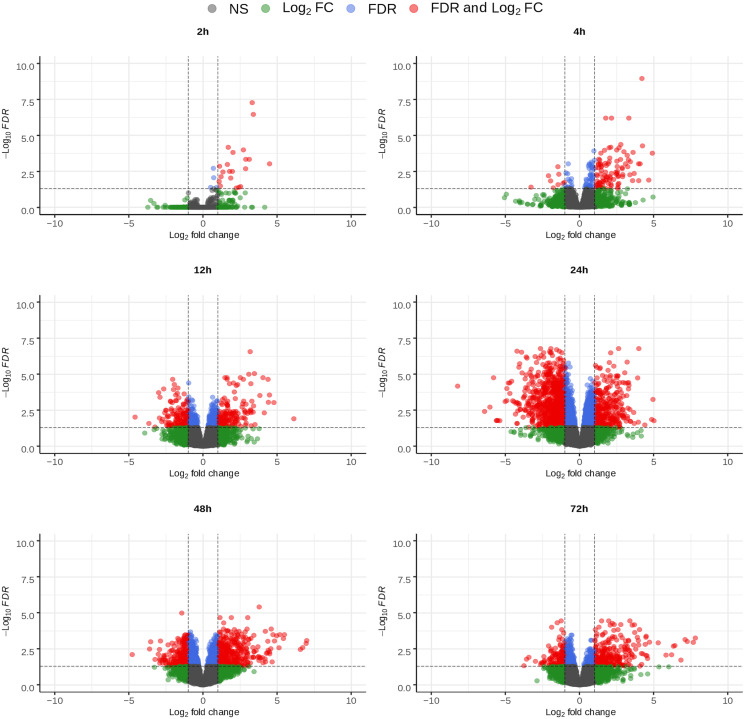
Volcano plots of the differential expression, mRNA from *E. tenella* infected *vs* uninfected cells, of all chicken genes at the indicated time points in mRNA samples from chicken HD11 cells infected *in vitro* with purified *E. tenella* sporozoites at 0 h. The significance thresholds were set at log_2_ fold change of ±1 and a false discovery rate of 0.05. NS stands for non-significant.

**Fig. 4.**
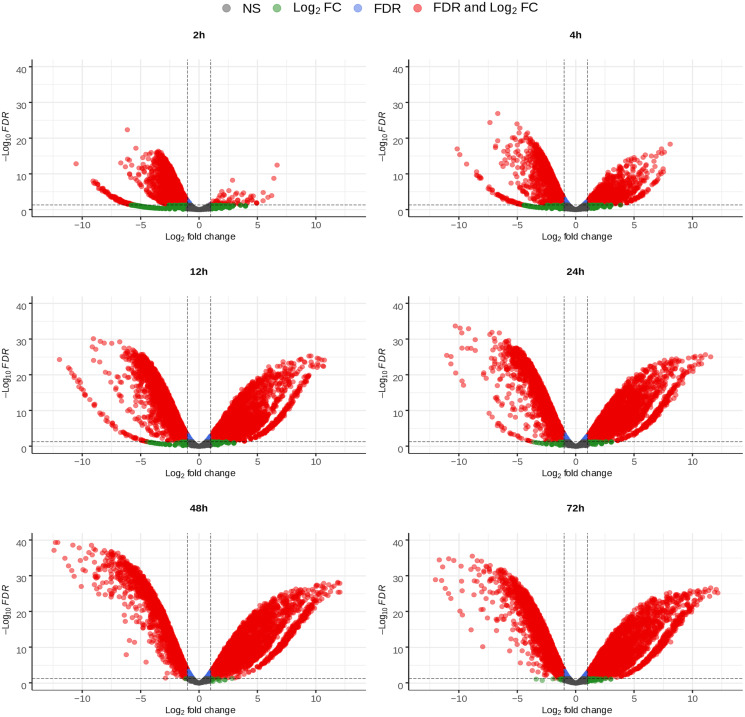
Volcano plots of the differential expression, *E. tenella* mRNA from infected HD11 cells *vs E. tenella* mRNA from sporozoites, of all *E. tenella* genes at the indicated time points in mRNA samples from chicken HD11 cells infected *in vitro* with purified *E. tenella* sporozoites at 0 h. The significance thresholds were set at log_2_ fold change of ±1 and a false discovery rate of 0.05. NS stands for non-significant.

DE analysis was conducted for chicken gene expression at each time point, comparing samples from infected cells to samples from uninfected ones collected at the same time point. For *E. tenella*, gene expression from each time point was instead compared to that in the pure sporozoite sample. The volcano plots (Figs 3 and 4) show the changing expression profile of each organism as the infection progressed. For the chicken (Fig. 3), the number of upregulated genes increased up to 24 hpi, where it peaked and then decreased slightly at 48 and 72 hpi. The number of downregulated genes increased in a slower fashion and peaked at 24 hpi. A far smaller number of genes were significantly downregulated in the last two time points. For *E. tenella* (Fig. 4), a large number of genes were either up- or downregulated across the different time points, with both categories quickly increasing from 2 hpi and showing similarities between 12 and 24 hpi and 48 and 72 hpi, respectively. A relatively small number of genes were differentially expressed at 2 hpi, showing that expression remained fairly similar to the sporozoites.

### GO and KEGG analysis

In order to elucidate the broad function of the differentially expressed genes in each organism, both GO category and KEGG pathway enrichment analyses were run on both datasets. The top 10 most significantly enriched categories at each time point can be found in Supplementary Tables S2–S5 with the main results presented here.

At the earliest two time points, the top GO categories for the chicken indicated a response to environmental stimuli, an upregulation of transcription and a general upregulation of metabolism. This includes categories such as ‘Response to chemical’ (GO: 0042221) and ‘Positive regulation of transcription by RNA polymerase II’ (GO: 0045944). At 12 hpi, the top categories had shifted towards signalling, such as ‘Cell communication’ (GO: 0007154), and developmental processes. For the earlier time points, the categories were generally upregulated but at 12 hpi there was a mix of up- and downregulated genes within each category. At 24 hpi, the largest number of significantly differentially expressed genes were observed, both up- and downregulated, the majority of which were involved in developmental processes. At 48 and 72 hpi, the top categories shifted back to cellular response, signalling and sensing but with more of a mix of up- and downregulation than in the early time points.

In the KEGG pathway enrichment for the chicken, a clearer pattern emerged. At 4 hpi, there was a general upregulation of a variety of signalling pathways, such as ‘NOD-like receptor signalling pathway’ (KO: 04621) and ‘Toll-like receptor signalling pathway’ (KO: 04620), likely to be partly due to shared genes across these pathways but also indicating a response to infection. The upregulation of signalling pathways continued at 12 hpi, although there were several downregulated genes within most pathways. A significant downregulation of the ‘Metabolic pathways’ (KO: 01100) also indicated that the metabolism of the cells was being affected. For the remaining time points, the ‘Lysosome’ (KO: 04142) and ‘Phagosome’ (KO: 04145) pathways were the two with most significantly altered expression. Within both pathways, genes were significantly downregulated, with the strongest downregulation at 24 hpi. The ‘Metabolic pathways’ remained significantly downregulated as a whole, but also several upregulated genes, indicating that the general metabolism was strongly affected by the infection. At 48 hpi and continuing at 72 hpi, signalling pathways returned to the top 10 most significant categories but the pathways that were upregulated during early time points were downregulated here.

For the *E. tenella* GO analysis, two categories were consistently affected across all time points: ‘Dephosphorylation’ (GO: 0016311) and ‘mRNA splicing *via* spliceosome’ (GO: 0000398), both significantly downregulated. Another category, ‘Translation’ (GO: 0006412), was significantly upregulated from 12 hpi onwards. More generally, a variety of RNA and DNA processing and regulation of processing categories were among the top significant categories, generally downregulated. At 12 hpi, ‘Glycolytic process’ (GO: 0006096) also became significantly upregulated and remained among the top categories from there onwards, likely due to the growth and replication occurring at the later time points.

For the KEGG pathways, the ‘Spliceosome’ (KO: 03040) pathway was the most significantly enriched at all time points, always downregulated. The ‘Ribosome’ (KO: 03010) and ‘Proteasome’ (KO: 03050) also appear at all but the first time point, always upregulated. Otherwise, there was a clearer signal from expression in metabolic gene categories at 12 hpi onwards, with both ‘Glycolysis / Gluconeogenesis’ (KO: 00010) and ‘Citrate cycle (TCA cycle)’ (KO: 00020) pathways being significantly upregulated, among others.

### Expression of chicken immune genes and *E. tenella* invasion/infection genes

**Fig. 5.**
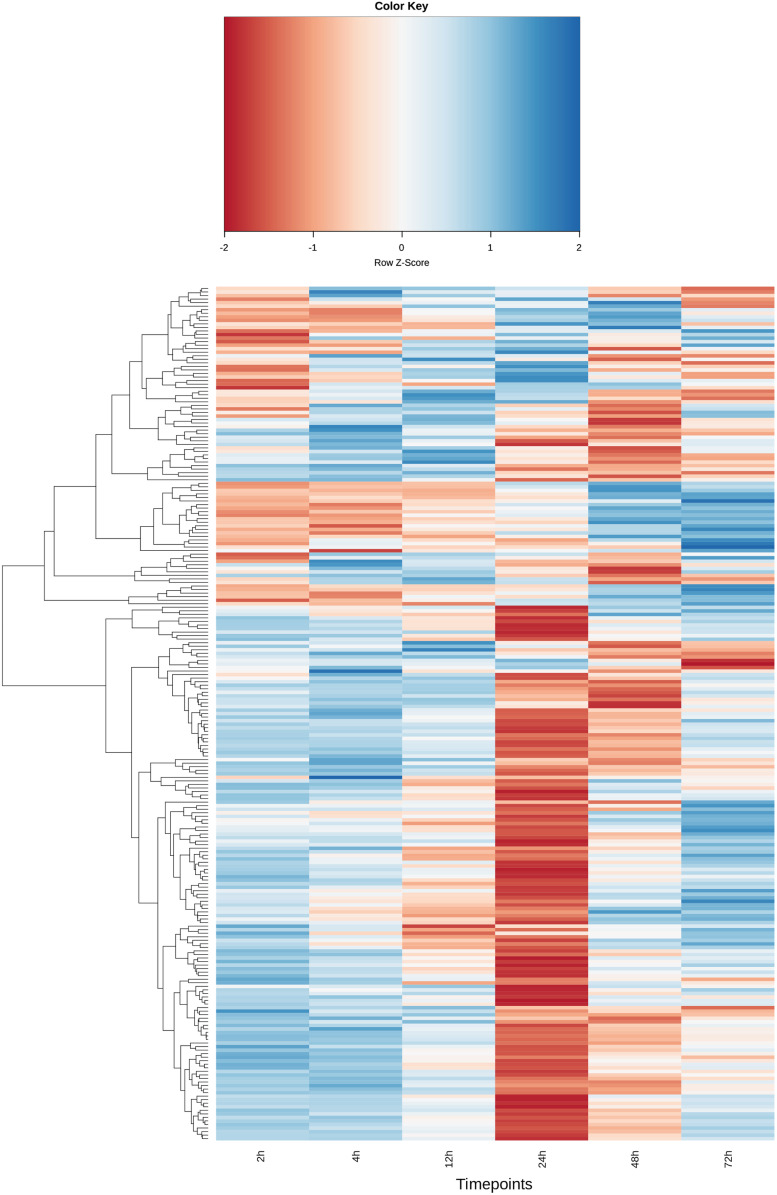
The heatmap depicts the expression profile of 241 immune-related chicken genes in mRNA samples from chicken HD11 cells infected *in vitro* with purified *E. tenella* sporozoites at 0 h. Blue represents upregulation and red downregulation. Expression is normalized within each row. For details on the selection of immune-related genes see Materials and Methods.

Separate analyses of genes putatively involved in host immune responses and parasite invasion/infection processes were also undertaken. In order to examine the immune response in the chicken cells, all genes that were associated with GO:0002376, ‘Immune systems process’ including all subcategories, as well as immune system-related KEGG pathways were identified. Of these genes, those that showed significant DE in at least one-time point were then plotted in a heatmap (Fig. 5) together with a hierarchical clustering dendrogram, showing which genes had similar expression patterns. This analysis showed a few dominant patterns of expression. A majority of genes were heavily downregulated at 24 hpi, especially those that were upregulated at 2 and 72 hpi. A smaller number of genes depicted at the top of Fig. 5 showed the opposite pattern.

**Fig. 6.**
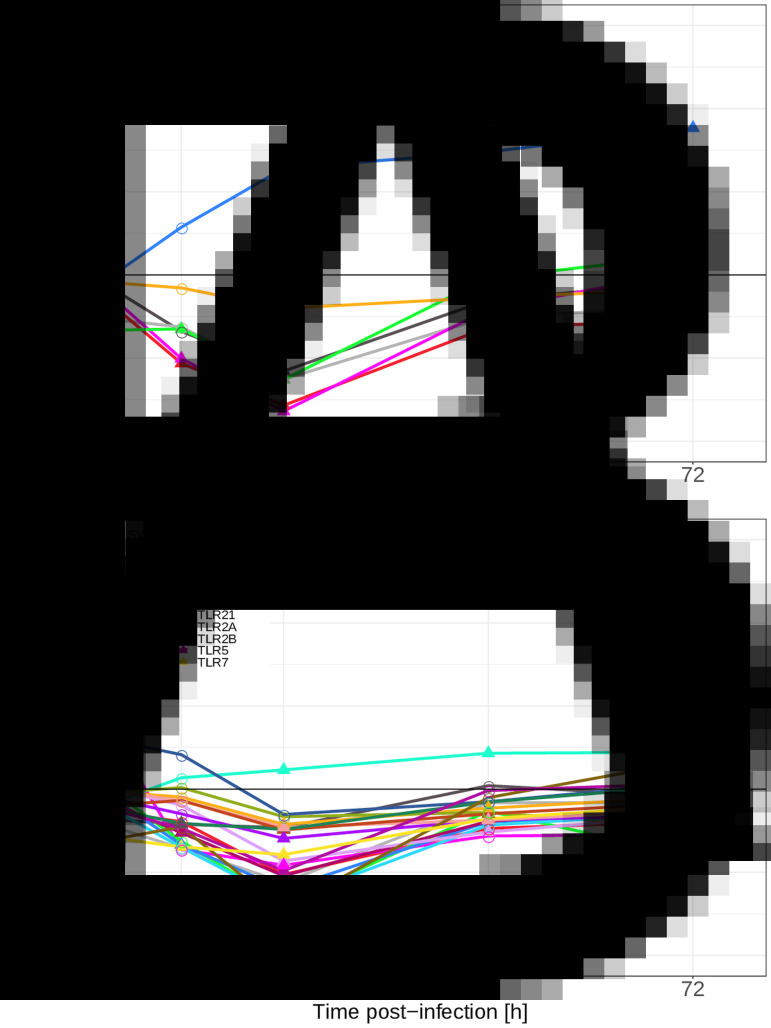
Differential expression, mRNA from *E. tenella* infected *vs* uninfected cells, of chicken (A) mannose receptors and (B) pattern recognition receptors in mRNA samples from chicken HD11 cells infected *in vitro* with purified *E. tenella* sporozoites at 0 h. Point shapes indicate significance, filled triangles for FDR < 0.05 and circles for FDR > 0.05, at the indicated time point.

In order to further elucidate the expressional patterns of specific host immune genes over the course of the infection, the profiles of some immune gene groups were manually curated and studied separately. Only genes that had an FDR <0.05 and a log_2_ fold change of at one or more in at least one time point were included in this analysis. Among these genes the mannose receptors (Fig. 6A) show a relatively clear pattern with all mannose receptors except for *MRC2* downregulated across all time points, most significantly at 24 hpi. On the contrary, *MRC2* was downregulated at 2 hpi but was subsequently increasingly upregulated with time, though not significantly so until at 48 and 72 hpi.

For the other PRR genes identified in this analysis (Fig. 6B) the majority were also downregulated with highest significance at 24 hpi. Two genes were significantly upregulated, *TLR15* at 4 hpi and *NLRC5* from 24 hpi onwards. *TLR15* was also upregulated, though non-significantly at 2 and 12 hpi and the same applied to *CLEC17A* at 4 hpi.

**Fig. 7.**
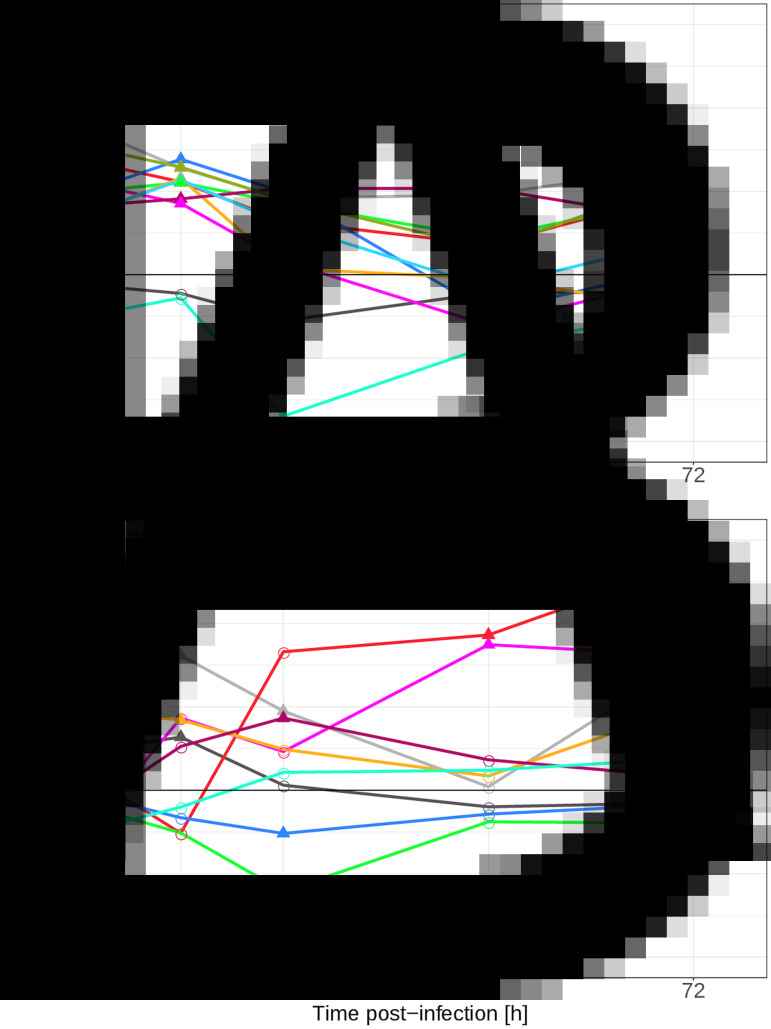
Differential expression, mRNA from *E. tenella* infected *vs* uninfected cells, of chicken (A) chemokines and (B) cytokines in mRNA samples from chicken HD11 cells infected *in vitro* with purified *E. tenella* sporozoites at 0 h. Point shapes indicate significance, filled triangles for FDR <0.05 and circles for FDR >0.05, at the indicated time point.

For chicken chemokines (Fig. 7A) a pattern of early upregulation, with most being significantly upregulated at 4 and 12 hpi was observed. Their expression then decreased and reached a minimum at 48 hpi and subsequently increased again at 72 hpi. A few chemokine genes did not follow this pattern: *CX3CL1* remained significantly upregulated from 4 hpi onwards and its expression only decreased at 72 hpi and *CCL1* had a similar pattern. *ATRN* and *CXCL12* were both downregulated across the time points, most significantly at 24 hpi. For the more heterogeneous group of chicken cytokines (Fig. 7B) a general expression pattern was not clearly observed. However, an early rise in significant upregulation could be observed in several genes with a peak at 12 hpi rather than at 4 hpi, as observed for the chemokines. This applied to *CSF1*, *CSF3* and *IL1B*. In contrast to most other immune-related genes, *TGFA* showed a peak of upregulation at 24 hpi. Finally, *IFNW1*, i.e. the gene for interferon-*β* (*IFN-β*) and *IL11* showed high and significant upregulation at 48 and 72 hpi but no significant DE at earlier time points.

For *E. tenella* we specifically studied genes with putative involvement in host cell invasion and infection such as SAG genes, rhoptry kinase (ROPK) genes, rhoptry neck protein (RON) genes, dense granule (GRA) genes and microneme (MIC) genes. All annotated genes associated with each of these categories in the *E. tenella* genome that had an FDR <0.05 and a log_2_ fold change of at least one in at least a single time point were included in the analysis.

**Fig. 8.**
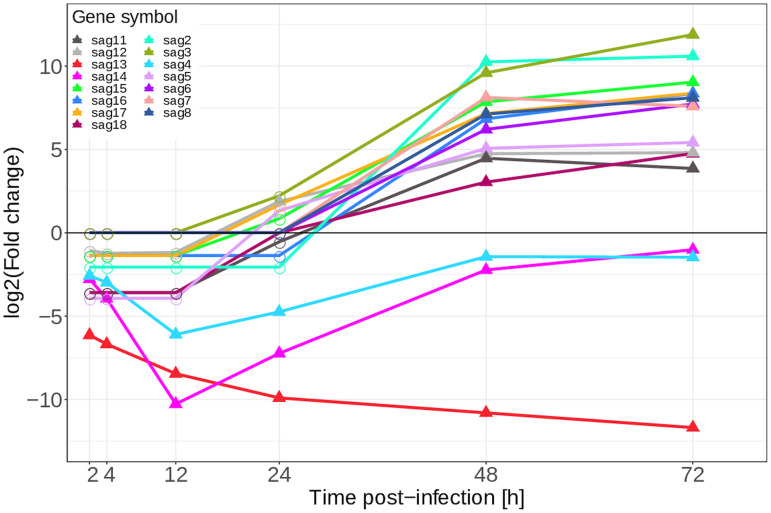
Differential expression, *E. tenella* glycosylphosphatidylinositol-anchored surface antigens mRNA from infected HD11 cells *vs E. tenella* mRNA from sporozoites, of *E. tenella* surface antigens in mRNA samples from chicken HD11 cells infected *in vitro* with purified *E. tenella* sporozoites at 0 h. Point shapes indicate significance, filled triangles for FDR <0.05 and circles for FDR >0.05, at the indicated time point.

Most of the SAG genes (Fig. 8) showed a consistent pattern: non-significance until 48 hpi, at which point they were strongly and significantly upregulated, remaining at a similar level at 72 hpi. In contrast, *SAG13*, *SAG14* and *SAG4* were downregulated across all time points.

**Fig. 9.**
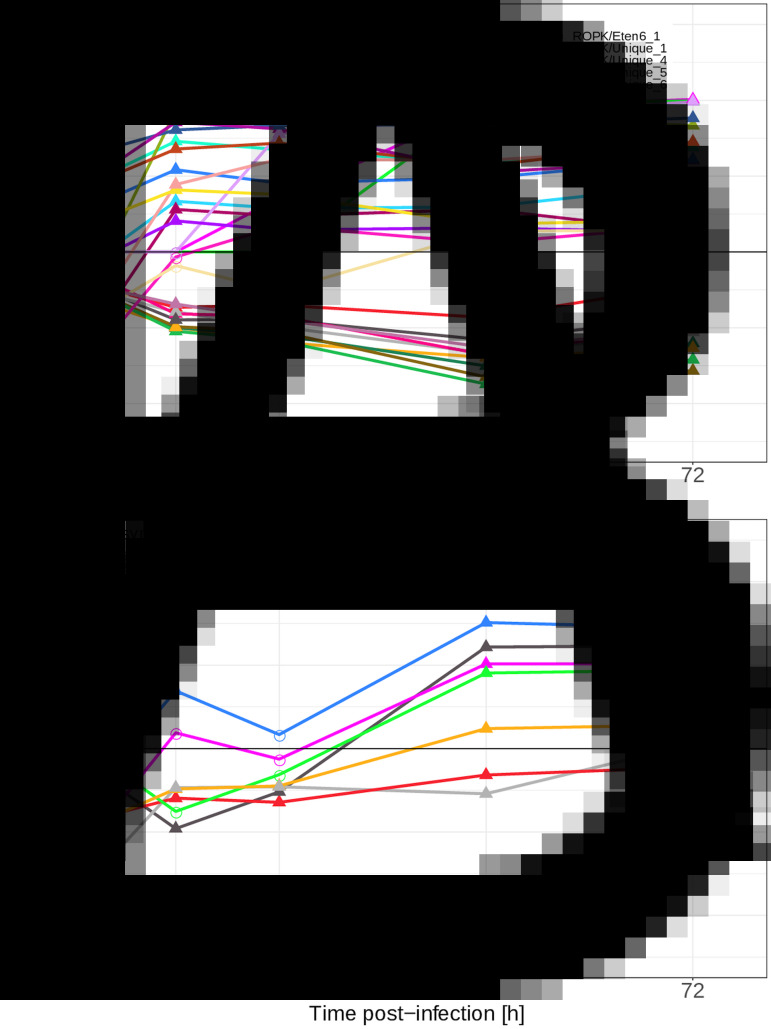
Differential expression, *E. tenella* mRNA from infected HD11 cells *vs E. tenella* mRNA from sporozoites, of *E. tenella* (A) rhoptry kinases and (B) rhoptry neck proteins in mRNA samples from chicken HD11 cells infected *in vitro* with purified *E. tenella* sporozoites at 0 h. Point shapes indicate significance, filled triangles for FDR <0.05 and circles for FDR >0.05, at the indicated time point.

The rhoptry kinases were annotated according to the results of Talevich and Kannan (2013), who examined and annotated ROPK genes in several important Apicomplexans. Multiple genes belong to some of the annotated subfamilies and these are specified with arbitrary numbering at the end of the name. The ROPK genes in *E. tenella* (Fig. 9A) clustered in two distinct expression profiles: general upregulation, especially from 24 hpi and onwards, and general downregulation.

The RONs are another set of rhoptry proteins present in Apicomplexans, including *E. tenella* (Oakes *et al*., 2013). They were identified by taking the best hits from Oakes *et al*. (2013) for each gene. In the present study, these genes were generally upregulated at 48 and 72 hpi, with only *RON3L1* being downregulated across all time points (Fig. 9B). *RON8* and *RON4L1* had a significant peak in upregulation at 12 hpi. The overall pattern resembled that of the SAGs and MICs.

**Fig. 10.**
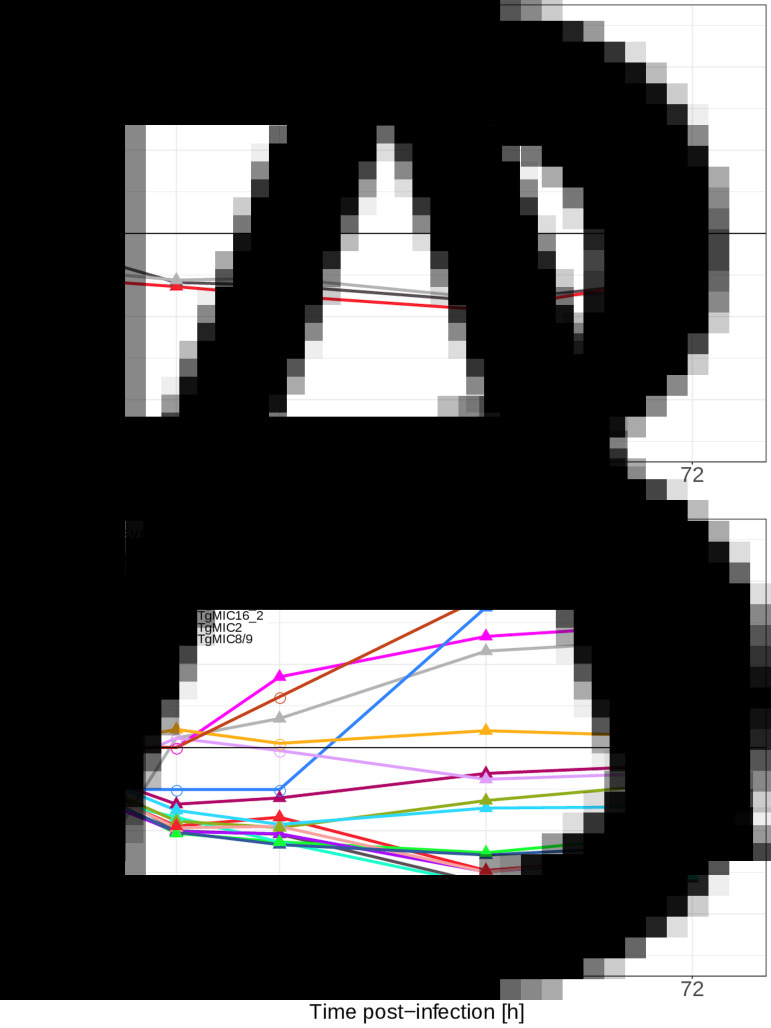
Differential expression, *E. tenella* mRNA from infected HD11 cells *vs E. tenella* mRNA from sporozoites, of *E. tenella* (A) dense granule genes and (B) micronemes in mRNA samples from chicken HD11 cells infected *in vitro* with purified *E. tenella* sporozoites at 0 h. Point shapes indicate significance, filled triangles for FDR <0.05 and circles for FDR >0.05, at the indicated time point.

Figure 10A and B show the expression patterns of *E. tenella* GRA genes and MIC genes, respectively. For *E. tenella* GRAs only three genes were identified here, only two GRAs were annotated in the *E. tenella* genome and only one significant homologue was found in the *T. gondii* genome. Our analysis showed that all GRAs were downregulated across time points (Fig. 10A). For MIC, several genes have been identified in *E. tenella*. Those with the Et prefix were annotated in the *E. tenella* genome but had no significant match in *T. gondii*, those with the Tg prefix were identified as homologues to *T.* gondii MIC genes but were not annotated as MIC genes in *E. tenella* and those without a prefix matched between the two organisms (Fig. 10B). Most of the MIC genes were downregulated across the time points. Four MIC genes were however significantly upregulated: *EtMIC3* and *TgMIC8/9* were both significantly upregulated at 48 and 72 hpi, *EtMIC13* was significantly upregulated from 24 hpi and onwards and *EtMIC 8* had a significant but low upregulation at 12, 48 and 72 hpi.

## Discussion

In the present study, host and gene transcription during the first merogony of *E. tenella* infection of chicken macrophages was monitored using dual RNA-Seq analysis. The read mapping step of the analysis elucidated that the fraction of reads mapping to features in the pure *E. tenella* sample was only ~65% compared to ~85% for the macrophage samples, while ~90% of reads mapped to the reference genomes in both cases. This indicates high levels of transcriptional activity in regions outside of annotated features in the *E. tenella* genome. A possible explanation for this lies in the *E. tenella* reference genome (GCF_000499545.2), which is fragmented into over 4000 contigs. The structural annotation covers over 8600 genes, only a small fraction of which has annotated alternative splicing. Functionally, the annotation is also relatively poor, with a large fraction of genes being annotated with a generic function or as hypothetical. With such a relatively poor annotation, it remains possible that there are exons or entire genes missing. It is therefore imperative that the structural annotation of the *E. tenella* reference genome is improved in order to facilitate further research. The data from this study can be useful for such efforts, especially as it is collected across multiple stages of the infection process. The functional annotation also needs improvement in order to allow for a more accurate interpretation of results from transcriptional experiments. Studies such as the one by Talevich and Kannan (2013), where they improved the annotation of ROPK genes in the *E. tenella* genome by building a general HMM profile of ROPK genes in Apicomplexans and used it to identify novel ones in both *E. tenella* and other Apicomplexans, show that this is quite feasible. Long-read technology may aid in building a less fragmented reference genome, which would also aid in improving the reference genome.

For the *E. tenella* reads from the infected macrophage samples, the fraction of these reads varied greatly between samples, both in time and within each time point between the biological replicates. As expected, the early samples contained a relatively small fraction of *E. tenella* reads, which grew as the parasite began to replicate. The variance of the fraction of *E. tenella* reads in each time point also differed. It appears to be similar for the samples from 4, 24, 48 and 72 hpi whereas the ones from 2 and 12 hpi show barely any variance.

Given that only a small fraction of each infected sample was made up of reads from the *E. tenella*, considerably deeper sequencing was needed compared to standard RNA-Seq in order to achieve a good representation of the parasite's gene expression. The produced depth ranged from 10 to 35 million reads, with most samples having a depth of 10–20 million reads. However, at some of the early time points, only >10^5^ reads were from *E. tenella* giving less data on the earliest behaviour of the parasite and potentially missing low-expressed genes. A similar study carried out on the *T. gondii* infection of porcine kidney cells had about twice the read depth used here but also had a larger fraction of parasite reads, especially at later time points, giving more information on parasite expression (Zhou *et al*., 2016). This showcases the importance of pilot studies to gauge the required depth of sequencing at each time point while keeping costs down.

The GO category and KEGG pathway enrichment analysis indicated an overall upregulation of signalling pathways in the chicken macrophage cells at 2–4 hpi and downregulation at 48–72 hpi. A mix of up- and downregulation was observed for metabolic pathways at 12–72 hpi. Of particular note here are the ‘Lysosome’ and ‘Phagosome’ KEGG pathways, which were strongly downregulated from 12 hpi onwards. These pathways are important in autophagy, which has been shown to have a role in the immune response against other Apicomplexans, for example against *T. gondii* in humans (Krishnamurthy *et al*., 2017). Their downregulation here may indicate that they also have an important role in the immune reaction to *E. tenella* and that the parasite may be defending itself by causing a downregulation of these pathways.

In *E. tenella*, the downregulation of various DNA and RNA processing categories was prevalent across the different time points. Of particular note is the ‘Spliceosome’ KEGG pathway, highly downregulated across all time points, and the ‘Ribosome’ and ‘Proteasome’ pathways, highly upregulated across all but the first time point. These same pathways, except for ‘Proteasome’, are also among the most significantly enriched in a comparison of merozoites and sporulated oocysts in *E. maxima* (Hu *et al*., 2018), indicating that a lower level of splicing and increased protein expression may be a general feature of *Eimeria* merozoites compared to sporozoites. The GO categories tell much the same story, with the downregulation of ‘mRNA splicing, *via* spliceosome’ and upregulation of ‘translation’. The second pattern of interest is that from 12 hpi onwards, categories associated with energy metabolism are upregulated. This coincides with a growth in the fraction of parasite reads and therefore likely linked with trophozoite formation and growth and the asexual replication phase of the first merogony.

Recognition of infectious agents by PRR is an important step in the initiation of effective immune responses to infection. In the present study we found that the genes of several PRR were differentially expressed during the *E. tenella* infection and three PRR stand out in our analysis; *MRC2* (also known as *uPARAP/Endo180* or *CD280*), *TLR15* and *NLRC5*. Among the differentially expressed genes of the mannose receptor family, all except *MRC2* shared a common expression profile with significant downregulation at 24 hpi, while *MRC2* was progressively upregulated throughout the experiment. In mammals, it has been shown that the ligand for MRC2 is collagen and the receptor has a role in collagen turnover (Melander *et al*., 2015). Interestingly, *MRC2* gene expression has also been shown to increase in spleen cells of mice after infection with *Plasmodium* spp (Rosanas-Urgell *et al*., 2012). Thus, it is possible that MRC2 is involved in the response to Apicomplexan parasites in both birds and mammals.

Most other differentially expressed genes with PRR functions identified in the present study also showed a downregulated pattern with maximal downregulation at 24 hpi. However, *TLR15* was an exception to this and showed upregulation during the early infection, 2–12 hpi, with peak expression at 4 hpi. TLR15 is unique for the avian and reptile lineages and related to the mammalian TLR2 family but distinct from avian TLR2 (Boyd *et al*., 2012; Oven *et al*., 2013). Interestingly, in mammals, TLR2 is one of the TLRs involved in the recognition of protozoan parasites and GPI moieties of parasite SAGs have been identified as TLR2 ligands (Egan *et al*., 2009; Denkers, 2010; Ghosh and Stumhofer, 2013; Dos-Santos *et al*., 2016). Activation of *TLR15* expression has been shown upon stimulation with different organisms including *E. tenella* (Zhou *et al*., 2013) and some lipopeptide and yeast-derived agonists have been suggested (Boyd *et al*., 2012; Oven *et al*., 2013). Thus, it is possible that an *E. tenella* SAG will be identified as a TLR15 ligand in the future.

Moreover, expression of *NLRC5*, a member of the CARD domain-containing, nucleotide-binding oligomerisation-like receptor (NLR) family, progressively increased during the *E. tenella* infection with similar kinetics as *MRC2*. In mammals this cytoplasmic receptor is known to regulate MHC I expression but also suggested to contribute to immune responses in other ways, e.g. through regulation of type I IFN responses (Benkö *et al*., 2017). In chicken cells, *NLRC5* expression has been induced upon LPS stimulation (Ciraci *et al*., 2010) and infection with avian influenza virus (Chothe *et al*., 2020). It has also been suggested that *NLRC5* expression promotes type I IFN expression in chicken cells (Lian *et al*., 2012) but also, somewhat conflicting, the expression is also suggested to promote avian influenza virus (Chothe *et al*., 2020) and avian leukosis virus (Qiu *et al*., 2016) replication, respectively. Considering *E. tenella* causes an intracellular infection it is not surprising that a cytoplasmic PRR such as NLRC5 is involved in its recognition. Interestingly we also observe an increased expression of *IFN-β* concurrent with the increased *NLRC5* expression, which supports the earlier observation that NLRC5 could be a positive modulator of type I IFN expression in HD11 cells (Lian *et al*., 2012).

In the present study, *E. tenella* infection elicited a prompt expression of a number of chemokines and pro-inflammatory cytokines by the macrophages. This is an expected reaction upon infection and responses with several of these chemokines and cytokines have also been monitored upon *Eimeria* infection in chickens, e.g. *CCL4*, *IL-8*, *IL-1β* and *CSF3* (Laurent *et al*., 2001; Hong *et al*., 2006*a*, 2006*b*; Cornelissen *et al*., 2009; Zhang *et al*., 2017). A striking observation particularly for the cytokine responses but also many chemokines and many of the other immune system-related genes was the strong downregulation of expression at 48 hpi. This may be a physiological response by the host cells to avoid the negative effects of prolonged inflammatory reactions or due to immune evasive mechanisms exerted by the parasite. For related Apicomplexan parasite *T. gondii* it has been shown that several parasite proteins, e.g. some of the ROPK proteins, downregulate and/or modulate the host immune response in its own favour (Kemp *et al*., 2013; Behnke *et al*., 2016; Hakimi *et al*., 2017). In the current study, we observed upregulation of many *E. tenella ROPK*s during the early infection, some of which could consequently be involved in parasite immune evasion.

Two of the cytokines did not follow the common pattern with downregulation at 48 hpi; *IFN-β* and *IL-11*. Interferon-*β* is a type I IFN important in the innate response to intracellular pathogens and in regulating ensuing T-cell responses towards Th1-type responses and e.g. enabling cross-presentation by antigen-presenting cells to activate cytotoxic T-cells (CTL) (Le Bon and Tough, 2008; Sebina and Haque, 2018). Thus, since a Th1-type response comprising CD8+ T-cells of potential CTL phenotype is crucial for immunity against *Eimeria* infections (McDonald and Shirley, 2009; Kim *et al*., 2019; Soutter *et al*., 2020) the observed *IFN-β* expression may be important for regulation of such responses. Expression of another type I IFN, IFN-*α*, has also been observed after *Eimeria* infection of chickens (Hong *et al*., 2006*a*; Kim *et al*., 2008). Interleukin-11 on the other hand is a cytokine primarily associated with downregulation of pro-inflammatory responses and inhibiting Th1-type responses (Truong *et al*., 2018). Its expression could hence be involved in the observed downregulation of immune responses, initiated either by the host or the parasite.

Several categories of genes known to have important roles in infection in both *E. tenella* and *T. gondii* were examined. The first of these categories of genes were the SAGs, 23 of which are annotated in the *E. tenella* genome with several other candidate SAGs having been identified (Tabarés *et al*., 2004). SAGs 2, 4 and 12 have been shown to have a role in modulating the chicken immune response by affecting the expression of several cytokines, including IFN-*γ* and IL-10, while many of the others have been shown to be immunogenic (Chow *et al*., 2011). In the present study, most of the SAGs show significant and high upregulation at 48 and 72 hpi, indicating a role in the merozoites. This includes both *SAG2* and *SAG12*. However, *SAG4*, along with *SAG13* and *SAG14*, instead shows consistent and significant downregulation. These results are largely similar to those of Tabarés *et al*. (2004), with most SAGs being expressed in the merozoite rather than in the sporozoite. As these are surface proteins, it is highly plausible that many of them are expressed during the maturation of the sporozoites in the oocyst. A potential example to the contrary is *SAG13*, as this gene is expressed in both the merozoites and the sporozoites but a much higher level in the sporozoites. This is also in line with the results of Tabarés *et al*. (2004) who observed the expression of *SAG13* in both stages.

For *E. tenella* a large number of ROPK family members have been identified including several sub-families unique to *E. tenella* (Talevich and Kannan, 2013). In the present study, the ROPK genes showed varied expression profiles, even within sub-families. A good example is *ROPK/Eten_5*, where two genes were downregulated across all time points while the other two were upregulated, though at differing levels. Those ROPK genes that were generally upregulated appear to have a role in the merozoite stage of the *E. tenella* life cycle, potentially showing a difference in the infection mechanisms between sporozoites and merozoites. Indeed, several putative ROPK genes have been detected only in *E. tenella* merozoites rather than sporozoites, including ETH_00000075 (*ROPK/Eten_4*) and ETH_00005905 (*ROP35*) (Oakes *et al*., 2013). Some research has been done on the roles of ROPKs in *E. tenella*, for example, ETH_00005190 (ROPK/Unique_3) has recently been shown to have an important role in preventing host cell apoptosis and arresting the cell cycle (Diallo *et al*., 2019). However, this gene showed no significant expression during the current experiment, which may indicate that it is expressed earlier in the development of the sporozoite. For genes of the second set of rhoptry proteins, i.e. RONs we observed that *RON2*, *RON8*, *RON4L1* and *RON6* seemed to be expressed during merozoite formation. Indeed, RON2 and RON8 have both been isolated from merozoites while RON4L1 has not (Oakes *et al*., 2013), possibly indicating a difference between expression and translation patterns.

For expression of MIC protein genes of, i.e. MIC, we found a similar pattern to the other *E. tenella* genes, some were strongly downregulated across time points such as two versions of *AMA1*, and others appeared to be focused in the merozoites, such as *AMA1_iso1* and *EtMIC3*. Interestingly, EtMIC3 has been pointed out as important for *E. tenella* tissue specificity to the caecum (Li *et al*., 2020). In the case of the AMA1 paralogues, *AMA1* and *AMA1_iso2* both were expressed across all time points in the present study, though downregulated, while *AMA1_iso1* was almost exclusively expressed in the late time points. This may indicate that different versions of AMA1 are being used for different zoite stages.

In this study dual RNA-Seq allowed us to obtain a comprehensive view of the early interaction between *E. tenella* and the chicken host cell. For this initial analysis, we chose an *in vitro* system with a single host cell type. *In vitro* systems have drawbacks but may achieve valuable data that provide a foundation for better *in vivo* studies. In the present system, the HD11 cells readily supported the first schizogony of *E. tenella* and despite potentially not being the first choice of the host cell for the parasite macrophages have nevertheless been suggested to have a role in *Eimeria* infections *in vivo* (Van Doorninck and Becker, 1957; Trout and Lillehoi, 1993) and also primary chicken macrophages support *E. tenella* replication (Long and Rose, 1976; Zhang *et al*., 2018). Taken together our results provide new insights into host and parasite gene expression and suggest e.g. parasite genes of importance in the early infection events, pathways of recognition of *Eimeria* infection and distinct chemokine and cytokine profiles of the chicken immune system. This is valuable information for further *in vivo* studies on the early recognition of *E. tenella* infection.

## Data

Sequencing data were deposited in the Gene Expression Omnibus under accession number GSE154393 and the Sequence Read Archive under accession number SRP271757.

## Supplementary material

For supplementary material accompanying this paper visit https://doi.org/10.1017/S0031182021000111. click here to view supplementary material
